# Agarose and Its Derivatives as Supports for Enzyme Immobilization

**DOI:** 10.3390/molecules21111577

**Published:** 2016-11-19

**Authors:** Paolo Zucca, Roberto Fernandez-Lafuente, Enrico Sanjust

**Affiliations:** 1 Dipartimento di Scienze Biomediche, Università di Cagliari, 09042 Monserrato (CA), Italy; pzucca@unica.it; 2 Departamento de Biocatalisis, ICP-CSIC; C/Marie Curie 2, Campus UAM-CSIC, Madrid 28049, Spain; rfl@icp.csic.es

**Keywords:** enzymes, immobilization, stabilization, agar-agar, agarose, cross-linking, functionalization

## Abstract

Agarose is a polysaccharide obtained from some seaweeds, with a quite particular structure that allows spontaneous gelation. Agarose-based beads are highly porous, mechanically resistant, chemically and physically inert, and sharply hydrophilic. These features—that could be further improved by means of covalent cross-linking—render them particularly suitable for enzyme immobilization with a wide range of derivatization methods taking advantage of chemical modification of a fraction of the polymer hydroxyls. The main properties of the polymer are described here, followed by a review of cross-linking and derivatization methods. Some recent, innovative procedures to optimize the catalytic activity and operational stability of the obtained preparations are also described, together with multi-enzyme immobilized systems and the main guidelines to exploit their performances.

## 1. Enzyme Immobilization: An Overview

As biological catalysts, enzymes are biological macromolecules able to increase the rate of biochemical reactions without changing the reaction equilibrium [1]. Their distinctive features are very intriguing from the perspective of the economical effectiveness of large scale processes, drawing the interest of various industrial sectors. Enzymes are, in fact, very efficient catalysts, operating under mild conditions (aqueous environment, physiological pH, ambient temperature/pressure), and performing very precise reactions due to their outstanding chemo-, stereo- or regio-specificity and selectivity [1,2,3,4,5].

Accordingly, enzymatic large scale processes usually feature lower demands both in terms of cost and time [2,6], allowing enzymes to find applications in several fields, such as biosensor production [7], detoxification of pollutants [8,9,10,11,12], production of biofuels and other bioproducts [13,14,15,16,17,18], and the food and pharmaceutical industries [4,19].

However, the use of enzymes in homogenous catalysis suffers from numerous limitations hampering the economic feasibility of the processes [20]. The large scale production of enzymes, for instance, is very costly, and they are usually rather unstable. Moreover, after the reaction, soluble enzymes contaminate the reaction products, since their recovery is very challenging and expensive.

Many of these drawbacks can be overcome by making the enzyme insoluble in the reaction medium. The term “enzymatic immobilization” refers to the numerous techniques aimed to attach enzymes on solid matrices, retaining at least part of their catalytic activity [21,22,23,24].

Despite the additional costs related to the developments of the process, heterogenized enzymes overshadow the use of their soluble native forms for several reasons [25,26,27,28]. The recovery of the catalyst after the reaction, for instance, is efficient and immediate. Contamination of the products is minimized, and the enzymatic catalytic activity can be completely exploited in multiple catalytic cycles. In particular, enzyme recycle alone is capable of decidedly driving economical balance towards heterogenized enzymatic preparations. These outcomes dramatically affect the economic impact of a large scale process. Storage and operational stabilization is achieved in many instances, by preventing intermolecular interactions (proteolysis, interactions with gas bubbles), or rigidification (via multipoint covalent attachment) [29,30,31,32,33] also preventing subunit dissociation [34]. Besides, improvements of enzyme specificity and selectivity have been in some instances described [29,35]. Immobilization may be used even to purify the target enzyme, saving time and effort [36]. Furthermore, immobilized enzymes can be employed in wider ranges of pH values, temperature, presence of organic solvents, and even inhibition could be prevented or at least reduced [29,35,37]. On the whole, all these features allow to achieve higher productivity during industrial processes [38].

**Figure 1:**
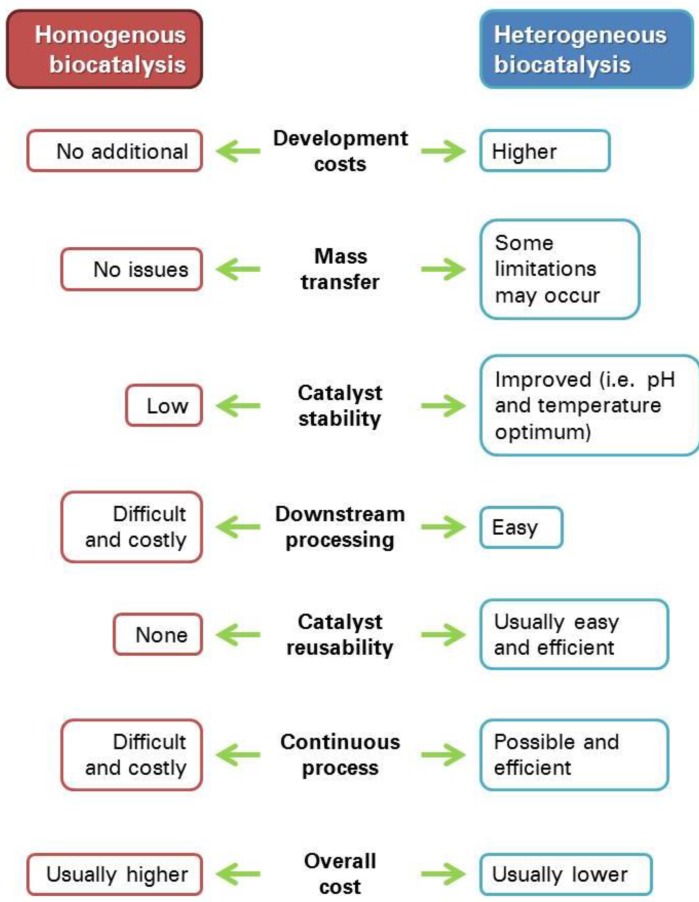
Comparison of main properties of enzymatic industrial processes, both in homogeneous and heterogeneous (immobilized) form.

Besides, soluble enzymes can be used for continuous industrial processes only under quite complex and expensive conditions (i.e., using membrane reactors), whereas their heterogenized counterparts are the ideal choices for large scale continuous processes [1]. The overall differences between homogenous and heterogeneous enzymatic catalysis are encompassed in Figure 1.

### 1.1. Choice of the Support

During the development of an immobilization process, the first choice is the selection of a proper solid support. Various requirements should be fulfilled to design a cost-effective protocol [39,40].

- Firstly, the costs of the unprocessed materials (both support and reagents needed for its possible functionalization) should be minimized.
- Supports and reagents should be harmless from both health and environmental perspective.
- Chemical and microbial inertness is usually a desirable feature.
- Mechanical properties should be compatible with practical applications.
- High surface area, large porosity and adequate particle size are basic requirements to be checked when selecting a matrix for an immobilization process.

Since enzymes are usually unstable in hydrophobic environments, hydrophilic materials form the best matrices for immobilization purposes. Moreover, inert supports should be preferred to prevent uncontrolled enzyme-support interactions [40].

Both organic and inorganic supports are commonly described in immobilization procedures [38]. The latter category includes many silica- and metal-oxide-based matrices such as zeolites, mesoporous silicas, alumina, ceramics, mesoporous glasses, magnetic nanoparticles [22]. They are all available with a great variety of prices and mechanical features [4,22]. The complete microbiological inertness and outstanding mechanical properties are their main distinctive traits.

There are several classes of organic supports, such as polyacrylamide-based and poly(vinyl alcohol) [41,42], polyamides (nylon, for instance [43]), and polysaccharides. Several macromolecular sugars find, in fact, application in the field of enzyme immobilization: starch, cellulose, but more recently also dextran, chitosan/chitin, alginate [44,45], and agarose derivatives have become widely popular [1,46].

Starch and cellulose are the most obvious choice from an economical perspective [39]. Unfortunately, starch is easily prone to microbial attack, making its use nowadays quite rare; moreover, starch-based supports show quite poor mechanical and rheological properties. Cellulose has a peculiar 3D structure that requires costly treatments to render a support suitable for immobilization techniques. In fact, cellulose chains form quite compact strands, linked each other by a regular set of interchain hydrogen bonds [47]. Such strands are impenetrable to most reagents, unless harsh treatments are applied. As an alternative, cellulose could be solubilized by suitable solvents, and then precipitated in an amorphous, swollen form, more accessible to derivatizing agents [48].

Other polysaccharides, such as dextrans and pullulan [49] in fact, are available in crosslinked forms quite resistant to bacterial/fungal degradation. Besides, these supports often present outstanding physical properties (such as, high surface area, and large pore size). However, dextran-derivatives (i.e., Sephadex^®^, and Sephacryl^®^) are quite expensive and their mechanical resistance is poor, with also poor geometrical congruence with enzymes. On the other side, agarose could be a natural alternative that in some forms could be more cost-effective: it is very hydrophilic, compatible with many activation strategies, readily available with a large variety of pore sizes and resistant to mechanical stirring. Moreover, the particle size may be modulated, from mm to µm range, depending on the final application.

### 1.2. Immobilization Techniques

**Figure 2:**
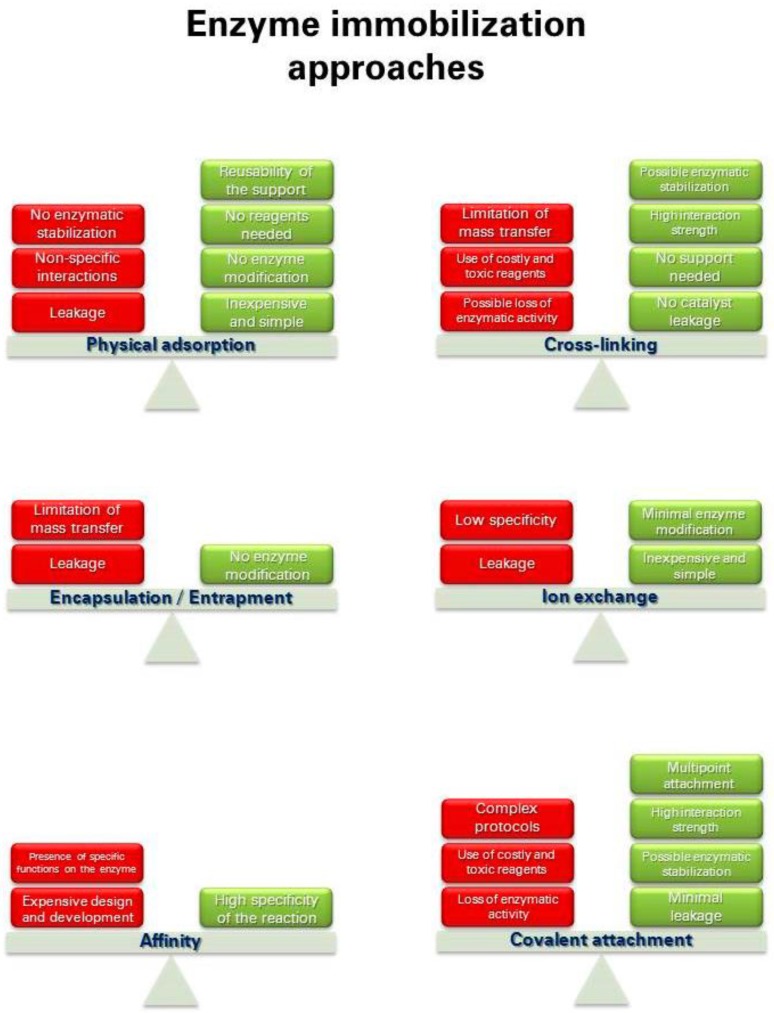
Main advantages and disadvantages of the most common immobilization techniques.

The first enzymatic immobilization was described in 1916, when Nelson and Griffin physically adsorbed invertase (E.C. 3.2.1.26) on charcoal [50]. Since then, in more than a century, several different approaches have been developed. Their main benefit and limitations are schematically reported in Figure 2.

Immobilization can involve a strong chemical bond between enzyme and support, a weak interaction, or no interaction at all. The latter includes the encapsulation and entrapment techniques, and is defined as the physical confinement of an enzyme within a 3D matrix network or membrane walls. Either gels or fibers, i.e., sol-gel matrices, are widely used in this approach, as protein diffusion is minimized. Enzymatic structure is usually not affected. On the contrary, mass limitation issues and enzyme leakage often occur [1,24,51,52].

The undesired protein release during reaction is the main drawback also when several weak physical interactions are involved in the enzyme immobilization, such as in the cases of adsorption and ion exchange. Several weak forces (i.e., van der Waals, hydrophobic, hydrogen bond, and ionic interaction) typically overlap in these approaches [23,53,54,55], leading to a type of interaction not specific or, at least, not predictable. On the bright side, these approaches are by far the simplest and the most inexpensive immobilization methods, not involving the use of supplemental chemicals [33].

Immobilization by affinity involves strong physical, but very specific interactions (i.e., ligand-protein), leading usually to a minimal leakage of active enzymes. Unfortunately, this procedure is different for each protein, causing a great economic effort for each design [56,57,58]. We should avoid involving the active center in the immobilization, and it is likely that distorted enzymes (after losing the affinity by the ligand) may be desorbed, contaminating the product.

Cross-linked enzymes (CLEs) (cross-linked enzyme crystals—CLECs, or cross-linked enzyme aggregates—CLEAs) are obtained by the reaction with bi- or multi-functional agents (such as glutaraldehyde oligomers or *bis*-imidoesters) attaching enzymes in large insoluble macro-aggregates, without the involvement of any support [4]. The strong interaction produces minimal enzyme leakage issues and in some cases operational stability increases, whereas toxic and costly reagents are required. The harsh operational conditions usually involved in cross-linking could also lead to protein modification and possibly inactivation. Diffusion problems and mechanical resistance are often problematic using these biocatalysts [33].

Immobilization by covalent attachment on preexisting supports assures the strongest interaction strength, minimizing leakage issues [22]. Several chemical approaches are known targeting different aminoacidic side chains, and requiring a strong background to be performed [22]. However, only with this technique can a stabilizing multipoint attachment be achieved. Immobilization by covalent attachment usually leads to massive structural modifications [1] even although enzyme activity may be quite preserved [59,60]. In some cases, this could negatively affect catalytic activity (for instance, by simple bad orientation of the enzyme active site). In other cases, on the contrary, the stiffening of tridimensional structure could enhance the enzymatic activity under some drastic conditions (such as extreme pH values, high temperature, presence of inhibitors or organic solvents) [61,62,63].

## 2. Agar-Agar and Agarose: Occurrence, Structures, Properties

Agar is a linear galactan polysaccharide extracted from some seaweeds belonging to the Rhodophyceae class [64,65,66]. It is presumably one of the first hydrophilic colloids discovered and purified [67]. A Japanese legend reports its discovery in the middle part of 17th century by the innkeeper Minoya Tarozaemon [66]. In 1658 the legend told the innkeeper received a Japanese officer and his party in a cold winter night. He prepared them a seaweed jelly dish by boiling *Gelidium* sp. in water. The surplus jelly was thrown away. During several nights and days, the jelly froze, thawed, and dried multiple times. Afterwards, Tarozaemon later took the white and dried residue, and found that the jelly could be remade by boiling with more water [67]. The method for agar production was thus accidentally discovered.

At first, agar was sold as an extract in solution (hot) or in gel form (cold), and was known as “*tokoroten*”. Since the 18th century agar has been used for food preparations (such as dessert gels, jellies, gelatin aspics, ice-creams, candies, canned meats, icings, and flan desserts [68]), and it has been commercialized in Japan as “*kanten*” (i.e., cold water), whereas in China has known as “*dongfen*” (frozen powder) [68]. The word “*agar*” (often doubled as “*agar-agar*”) is of Malaysian origin. In France and Portugal the terms “*gélose*” and “*gelosa*” are also used [66,67].

Agar was in Europe a couple of centuries after its discovery in Japan, and was being used as a food additive by the middle of the 19th century. In microbiology agar is widely used as the preferred solid culture media, since a very few microorganisms are able to hydrolyze its structure [69]. Although this application is traditionally attributed to Robert Koch in 1882 [70], it has been suggested that the idea actually came from his wife, Angelina, who firstly discovered the properties of agar during the preparation of jams and jellies [68]. It is a Generally Recognized as Safe (GRAS) food additive [64].

Agar is insoluble in cold water, but hydrates in boiling water. A 1.5% solution is clear and isotropic (usually it is difficult to obtain more than 6%–10% solutions even by autoclaving [39]). By cooling the solution below 35–45 °C, a firm and stable gel is obtained [66,71], stable up to 80–85 °C; gels can be obtained also when the agar concentration is as low as 0.1% [72].

Agar is extracted from the cell walls of some red seaweeds, belonging to Rhodophyceae class, widespread all along the world (including Japan, Korea, Spain, Portugal, some African countries, Mexico, Chile, and India). The genera *Gelidium* and *Gracilaria* in particular are the sources of a great part of the commercialized agar [64]. *Gelidium* species (such as *Gelidium amansii*, *Gelidium liatulum*, and *Gelidium pacificum*, see Table 1) were the traditional materials used in Japan, but shortages during World War II led to the introduction of *Gracilaria* species (which require previous alkali treatment to enhance gel strength [66,73]). *Gelidium* spp. produce the highest quality agar, but they are not abundant, and their cultivation is difficult. On the contrary, *Gracilaria* spp. are present in several countries, their cultivation is easier, and the agar production is less expensive [64]. Each year about 35,000–40,000 tons dry weight of *Gracilaria* are produced, and whereas the production of *Gelidium* spp. is below 20,000 tons [74]. The total annual production of agar from these starting materials is about 7500 tons.

As mentioned, the two agar sources sharply differ regarding the required pre-treatment. In the case of *Gelidium* the plant sample is diluted in a mild acid solution to improve the efficiency of the extraction, whereas, *Gracilaria* samples require a strong alkali solution treatment (2%–5% NaOH for 1 h at 90 °C) to convert sulfate groups to 3,6-anhydrogalactose [66]. In the absence of this treatment the mechanical properties of the obtained gels are too poor for practical applications.

### 2.1. Chemical Structure

**Figure 3:**
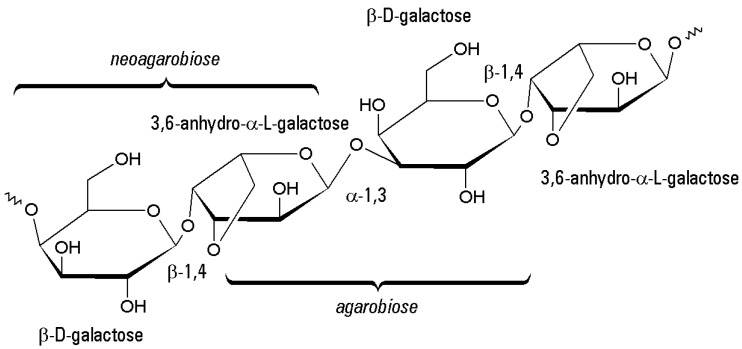
Backbone structure of agarose. The repeating disaccharide units are called agarobiose and neoagarobiose. In the case of agaropectin, 2 or 6 positions of 3,6-anhydro-α-l-galactose residues can be substituted by –OSO_3_^−^, –OCH_3_, glucuronate, or pyruvate residues [79,80].

Two main components can be identified in agar: agarose and agaropectin [65,75]. Agarose is a neutral gelling heteropolysaccharide, accounting for the major fraction of agar. It is a linear polymer with the repeating unit shown in Figure 3, containing both α- and β-glycosidic bonds (unlike the majority of the most common polysaccharides [76,77,78], including dextran). The two monosaccharide present are β-d-galactose and 3,6-anhydro-α-l-galactose, linked by glycosidic bonds β(1–4) (between β-d-galactose and 3,6-anhydro-α-l-galactose, giving the disaccharide basic unit called *neoagarobiose*) and α(1–3) (between 3,6-anhydro-α-l-galactose and β-d-galactose, giving the disaccharide basic unit called *agarobiose*).

These linkages can be hydrolyzed by two distinctive enzymes: α-agarase (E.C. 3.2.1.158) and β-agarase (E.C. 3.2.1.81) [64,81]. Only a few microorganisms have these enzymatic activities [69], explaining the wide use of agar in microbiological solid culture media.

Agarose presents a high degree of polymerization, since a molecular weight of at least 120,000 amu has been determined (corresponding to about 800 sugar residues [67]). Sulfate can be present only below 0.15%.

The rather heterogeneous agaropectin (or better agaropectins) has the same repeating unit [82], but about 8% of the 2- or 6- positions of the 3,6-anhydro-α-l-galactose residues can be substituted by –OSO_3_^−^, –OCH_3_, glucuronate, or pyruvate residues [79,80,83]. These substituents greatly affect the gelling potential of the material. Agaropectin shows a lower MW (about 126,000 amu, corresponding to less than 100 monosaccharide units).

Agarose polymers form tight bond trunks of large diameter, much larger than an enzyme, and the diameter increase when the percentage of agarose does; in that way a higher enzyme support/interaction may be obtained using agarose with large percentage. Moreover, the agarose percentage and loading capacity are positively correlated. However, the larger the agarose percentage is, the lower the pore diameter is. That way, optimization of the agarose used for each enzyme needs to be performed [84].

### 2.2. Functional Properties

In biochemistry agar and agarose gels have been used both as supports for electrophoresis [85], and for protein immobilization [86,87], because of some favorable functional properties. In water, agarose is a typical strongly hydrophilic, lyophilic and extremely inert colloid. Its ability to reversibly form stable and firm gels is the most appealing feature of agar and agarose.

In boiling water, in fact, agarose solutions have a random and fluctuating coil conformation. When the solutions are cooled below 35–45 °C, gelation occurs, since a rigid ordered structure is obtained with co-axial single and double helices formation [80]. These left-handed helices [88] are stabilized by the interaction with water inside the cavities, and by inter-chain interaction of hydroxyl groups, allowing the aggregation of up to 10,000 helices in spherical microdomains [66]. The transition is completely reversible [66], without modification of the mechanical properties of the gel, unless very low pH values (<4) or oxidizing agents are used. The process is totally dependent on hydrogen bond formation. Accordingly, chaotropic agents and proton scavengers (such as urea, guanidine, or thiocyanate) prevent gel formation by impeding the creation of the hydrogen bond network.

For a long time, agarose dispersions (“solutions”, agarose sols) in water have been regarded as formed by flexible, random-coiled chains [89] that gradually turn into single helices and finally to double helices, that further aggregate to form bundles. Such bundles present junction points or knots, leading to a typical 3D lattice, whose large pores host a lot of water molecules. Water is not merely contained in a ‘passive’ manner within the polymer pores, but on the contrary actively participates in gel stabilization by means of a complex net of hydrogen bonds [90]. However, another study suggested that the gelation process is rather governed by a single loose helix to single rigid helix transition [91]. The high turbidity shown by agarose gels also at low solid content (~1%) indicates the heterogeneous nature of the 3D network, which is formed by compact polymer bundles forming large cavities or pores containing water only [92,93]. Experiments performed in the presence of binary solvents (water plus different concentrations of selected sugars, such as sucrose) and optical, rheological, and mechanical measurements have led to the conclusion that the force responsible for the gelation phenomena, with its pronounced hysteresis behavior, depends exclusively from a complex and dense network of hydrogen bonds between polymer chains, and between polymer chains and water [94,95].

As underlined above, agar and agarose possess an outstanding hydrophilic nature, being ideal for enzyme immobilization (cf. Section 1.1). It is noteworthy that, contrary to other hydrophilic supports (such as dextran-based, polyacrylamide, and polyvinyl alcohol), agar and agarose gels do not appreciably shrink or swell upon the change of solvents [39]; the 3-D architecture of the polymer net remains almost unchanged when the water molecules, hosted within the gel pores, are driven out and substituted by other solvents such as acetone. Also, the very slow and low syneresis [96] exhibited by agarose gels is so small that pore diameter is practically unaffected. This invariance in structural parameters is a very useful feature when planning and applying chemical modification protocols, requiring non-aqueous conditions to be accomplished (vide infra): it is a crucial feature that allows to retain the same bed volume when water is (partially) displaced by organic solvents (immobilized enzymes usually show an increased tolerance to less polar environments in comparison with their soluble counterparts).

Agarose is also quite chemically stable under common operational conditions (pH > 3). In the presence of reducing agents or absence of O_2_, the cross-linked agar gels could be treated up to 120 °C with 10%–30% NaOH without destruction of the matrix [39]. The stability in acid environments is lower, but short treatments with 1 M HCl or 50% CH_3_COOH are possible. Also in this case, cross-linking enhances stability.

Biological resistance is remarkable for a natural polymer, being comparable to synthetic supports. Only a few microorganisms in fact produce hydrolases (agarases) able to break β(1–4) and α(1–3) linkages present in the agarose backbone [64,69]. Chemically cross-linked agars are not significantly attacked at all. Besides, as already stated, agar is considered a Generally Recognized as Safe (GRAS) food-grade additive (compare the requirements the ideal support for immobilization should have reported in Section 1.1) [64].

Two main parameters are used to define the suitability of agar/agarose gels to practical applications: the gel strength and the hysteresis.

Gel strength is usually measured by the Nikan-Sui method (or Kobe test). This method uses a cylindrical piston with 1 cm^2^ area. The gel strength is expressed as the necessary load to break in 20 s a standard 1% gel [66]. Typical agar/agarose gels present strength up to 800 g/cm^2^. Usually *Gelidium* agars have strength around 300–500 g/cm^2^. Whereas *Gracilaria* agars show lower values (50–300 g/cm^2^) reaching the same values of *Gelidium* after alkaline pre-treatment [67]. Gel strength can be increased by crosslinking with bifunctional agents, such as divinyl sulfone or glutaryl chloride (vide infra) [39].

Hysteresis is the difference between gelling and melting temperature. This value is usually higher than other gelling agents (such as kappa carrageenan), since usually at least 50 °C values are described. In fact gelling temperature is around 30 °C (up to 42 °C for *Gracilaria*). And melting does not occur below 80–90 °C for *Gelidium* to 76–92 °C for *Gracilaria* [66].

These properties are very useful in practical applications since a moderate increase in temperature does not affect the stability of the gel. This is not affected either by the presence of cations, such in other gelling materials. Besides, no additional reagents are needed to provide gelation, such as potassium ions or proteins (carrageenans), calcium or other cations (alginates) [67,71]. Neither high sugar concentrations nor acid pH are needed (contrary to pectins).

Suitability for immobilization applications is confirmed also by the ability of agar/agarose gels to forms derivatives. Each agarobiose unit has four alcoholic functions (Figure 3). Three of them are secondary, one primary (main target of derivatizations), showing the same chemical reactivity of other polymers such as cellulose, starch, dextran-based, polyvinyl alcohol and so on.

From an economical perspective, it should be considered that crude agar is a very inexpensive matrix, whereas both purification to obtain agarose and then cross-linking gradually increase the costs. However, the immobilization purposes do not always require a high degree of purification or cross-linking.

## 3. Agarose-Based Supports: To Cross-Link or Not to Cross-Link?

As reported in Section 2, agar could be considered as a mixture of two main components, namely agarose (lower sulfation/esterification, higher molecular weight) and agaropectin (higher sulfation/esterification, lower molecular weight). In fact, this distinction between two fractions is somewhat arbitrary, and a sharp boundary between them is not easily traced. Anyway, agarose has to be de-esterified for the majority of technological applications, so that four free hydroxyls per agarobiose unit become available: three on the d-galactose monomer, and one for the l-anhydrogalactose monomer. These hydroxyl groups are responsible for the sharp hydrophilic character of the polymer and also for its gelling properties. Gradual cooling to 35–40 °C of hot solutions (more precisely, sols) of agarose, containing at least 0.1% polysaccharide, spontaneously gives rise to physically stable, elastic gels. Despite of their extremely high water content, these gels are surprisingly stable and, as it has been underlined above, maintain their 3D structure even in the presence of organic solvents such as acetone. Therefore, these gels—unless they are heated and brought to fusion—usually do not require additional chemical cross-linking, and are ready for chemical functionalization reactions, suitable for enzyme covalent attachment. In the case of chemical modifications requiring non-aqueous reaction media, a previous dehydration upon treatment with a gradient of acetone or lower alcohols is easily accomplished, possibly followed by further drying in vacuo.

Agarose gels could be easily prepared in beaded forms such as microspheres whose size and porosity could be tuned by carefully controlling the preparation procedures; among these, spray or suspension gelation are the most popular [97,98,99]. More recently, the use of microporous membrane emulsification techniques has allowed the preparation of monodisperse microspheres, perfectly suitable for column operation [100,101]. Beaded agarose preparations in the form of microspheres of selected diameters and porosity are widely commercially available from a number of suppliers; Sepharose^®^ (Uppsala, Sweden) is one of the best known commercial products.

However, for certain applications, chemical cross-linking by means of bifunctional agents is advisable, and leads to some advantages: (i) the cross-linked gels become relatively insensitive to heating, as the rupture of the hydrogen bonds within the polymer bundles and within the joining regions are prevented by the covalent, thermally stable chemical linkages. Cross linked agarose particles become by this way suitable for operating with highly thermo-tolerant enzymes; (ii) the preparations could be sterilized by autoclave if necessary; (iii) cross-linked preparations are on the whole decidedly more resistant against physical, chemical, mechanical agents. Moreover, they become quite insensitive to microbial attack. In particular, cross-linked beaded agarose could be chemically modified under operative conditions that would destroy plain agarose particles by dissolving them.

Provided that cross-linking treatments could often be carried on in the presence of water, beaded agarose gel particles (readily obtained by cooling biphasic systems under controlled temperature and stirring conditions) are simply reacted with the cross-linking agent under proper experimental conditions, directly affording the desired product. Several bifunctional agents are well known as cross linkers for agarose beads: among these, epichlorohydrin [39], 2,3-dibromopropanol [102], divinyl sulfone [103,104], *bis*-oxiranes [105]. The common feature of the crosslinking reactions is that they afford very rigid networks, without affecting the porosity of the gels. This depends on the cross-linking reactions taking place within the polysaccharide bundles that are reinforced with respect to their not cross-linked counterparts. The effect can be further enhanced in the presence of kosmotropic salts that promote water expulsion from the bundles, rendering them more prone to cross-linking. Anyway, the choice of proper experimental conditions allows obtaining products that are at the same time cross-linked and functionalized, provided that a fraction of the bifunctional agent reacts only by using only one end, whereas the other remains active and potentially ready to react with proteins or with other molecules.

**Figure 4:**
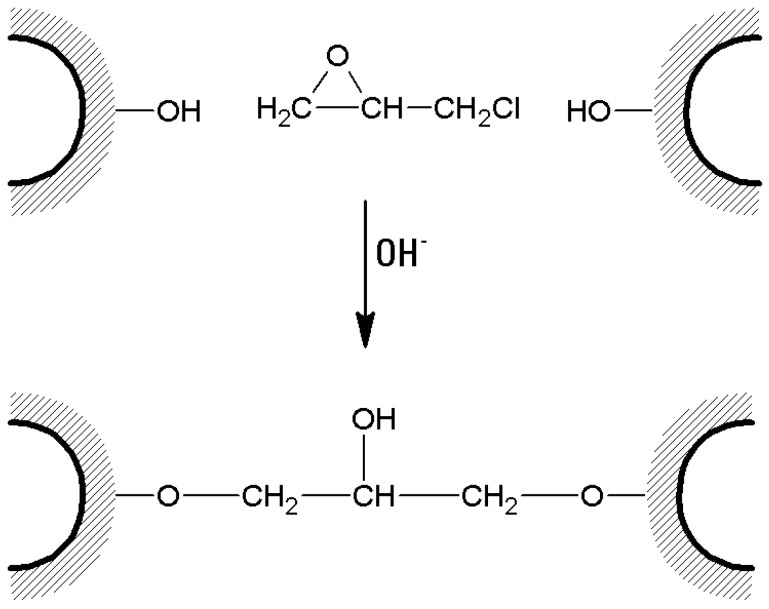
Cross-linking of agarose beads with epichlorohydrin.

Some examples follow, to depict general procedures to cross-link beaded agarose when required:

(a) A typical cross-linking procedure (Figure 4) involves the suspension of 1 L of swollen agar/agarose gel with 1 L NaOH 1 M, containing 100 mL epichlorohydrin and 5 g NaBH_4_. After 2 h of gentle stirring at 60 °C, the suspension is washed with hot water to neutrality, and re-suspended in 500 mL NaOH 2 M, containing 2.5 g NaBH_4_. The suspension is then treated at 120 °C for 1 h. Several washings are then performed, including 1.5 L of hot NaOH 1 M and 0.5% NaBH_4_, and 1.5 L of cold NaOH 1 M and 0.5% NaBH_4_. Then the suspension is cooled with ice and neutralized until pH 4 with CH_3_COOH [39]. The suspension can be stored in 0.02% sodium azide solution.

The only “cost” of this crosslinking step is the loss of the groups involved in the crosslinking for any further modification: the maximum activation of commercial cross-linked and not cross-linked agarose may differ in a 20%–30%. This may have some incidence in the final support-enzyme interactions [40].

(b) Beads prepared from agarose powder (6% *w*/*w*) are cross-linked with 1,3-dichloro-2-propanol (DCP) under strong alkaline conditions. A net weight of 1.5 g of beads is added to 10 mL of a solution of 0.3 M NaOH in distilled water, and the mixture treated with 0.1 mL of DCP. The reaction is allowed to proceed for 1 h, with continuous agitation at ~50 °C. After that, the beads are washed with water until the effluent becomes neutral [106].

**Figure 5:**
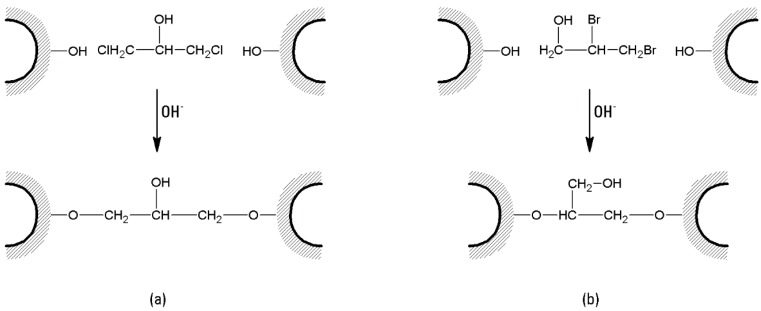
Cross-linking of agarose beads with 1,3-dichloro-2-propanol (DCP) (**a**) and 2,3-dibromopropanol (**b**).

A similar protocol can be employed in the case of 2,3-dibromopropanol (Figure 5).

**Figure 6:**
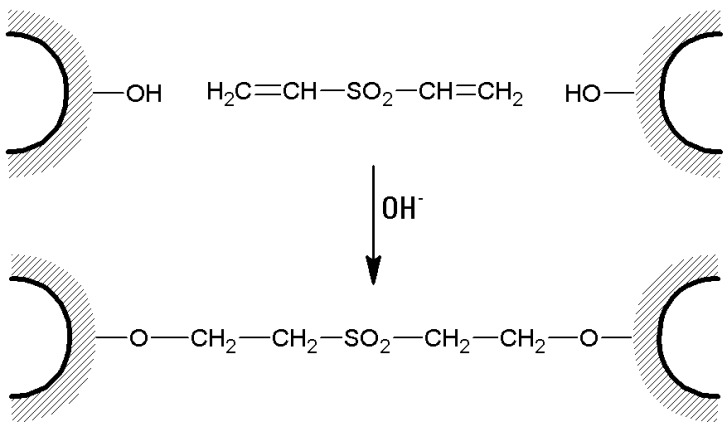
Cross-linking of agarose beads with divinylsulfone.

(c) Wet agarose gel (typically 10 or 20 g) is soaked with 10 to 20 mL of 0.5 M sodium carbonate buffer of pH 11. Then, divinylsulfone (DVS) is added in an amount expressed as % (*v*/*w*) of the wet gel, depending on the desired cross-linking degree, and the reaction is allowed to proceed for 2 h at room temperature (Figure 6). In fact, DVS influences gel stiffness and therefore water (or buffer) flow through a chromatographic column. The flow rate linearly increases as the cross-linking degree increases until about 3% cross-linking [103], which is the optimal choice to obtain rigid, stable, and fast-flow chromatographic media.

The gel is then washed with distilled water until it had neutral pH.

The DVS-treated gels contain unreacted vinyl groups, which can react with substances containing amino-, hydroxy- or mercapto-groups; the gel should be deactivated with such a substance. 2-Mercaptoethanol in neutral or slightly alkaline medium removes all vinyl groups that could be detected by titration with sodium thiosulfate solution. To *n* g of gel (wet weight) suspended in a total volume of 2*n* mL, 0.01*n* mL of 2-mercaptoethanol is added, and the reaction is allowed to proceed overnight; the excess of reagent is washed away with distilled water or neutral buffer [103].

Also amines or amine-containing compounds could be used under alkaline conditions; however, the introduction of electric charges on the gel should be taken into the due account.

**Figure 7:**
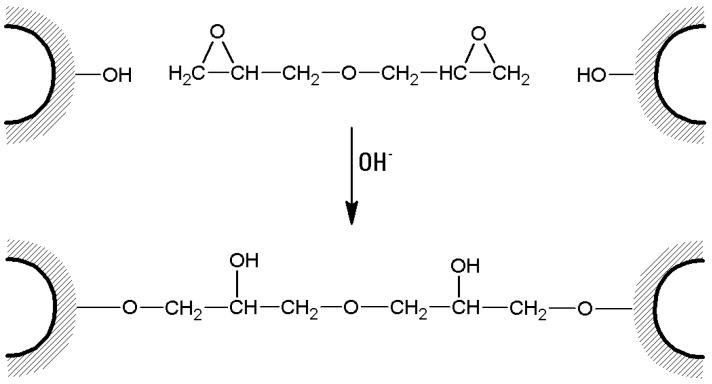
Cross-linking of agarose beads with a *bis*-oxirane (or *bis*-epoxide). Similar protocols can be performed with other cross-linking agent bearing longer spacers between the two epoxide functions.

(d) In the case of *bis*-oxiranes, 1 g of dried agarose reacts for 8 h at 25 °C with 1 mL of a proper cross-linker (i.e., diglycidyl ether) and 1 mL of NaOH 0.6 M (containing 0.2% *w*/*v* NaBH_4_) [105], as shown in Figure 7). Deactivation of unreacted oxirane groups can be performed by treatment of the gel with 2 M glycine or ethanolamine at pH > 8.5, 23 °C for 24 h.

## 4. Chemical Functionalization of Agarose-Based Supports: Chemistries and Protocols

Like other polysaccharides, the polyhydric character of agarose accounts for its reactivity: the hydroxyl functions could be partially or totally derivatized, affording ethers and esters with a vast variety of reagents. In this way, several new chemical functions can be grafted along the polymer chains, such as amine, carboxyl, sulfonate, cyano, dichlorotriazinyl, and so on. The unique gelling properties of agarose have been extensively studied for decades, and a reasonably deep knowledge has been reached about the features driving this crucial property.

Obviously, complete derivatization of hydroxyl groups is neither easy nor advisable: in fact, “internal” hydroxyls are poorly reactive towards chemical reagents for the reasons of sterical hindrance; moreover, a noticeable fraction of those hydroxyls are engaged in the gel network, and their modification would destroy the 3D structure causing the dissolution of the gel. Last but not least, excessive derivatization degree could deeply change the hydrophilic, protein-friendly character of plain agarose gels.

This said, several general methods, also suitable for polyhydric supports such as other polysaccharides and polyvinyl alcohol, work well on agarose gels. We will cite here some examples to produce agarose supports with special interest for enzyme immobilization.

**Figure 8:**
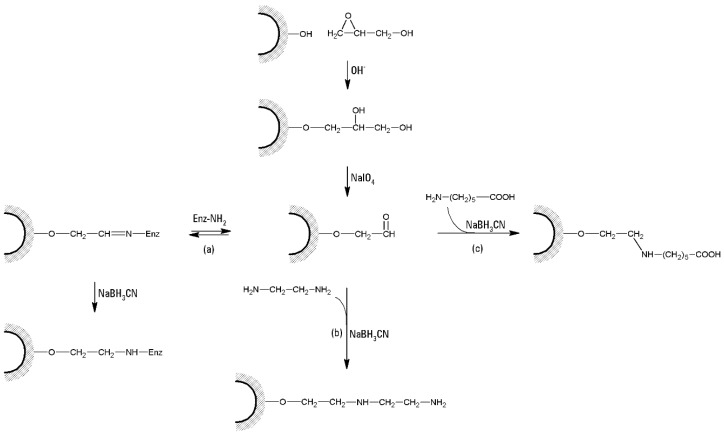
Preparation and application of glyoxyl agarose. This can directly couple with enzymes aminogroups, path (**a**). Alternatively, glyoxyl agarose can be used for the insertion on the support of amino-, path (**b**) (agarose-MANE), or carboxy-groups, path (**c**), that can in turn be used for other covalent immobilization procedure (i.e., carbodimide or glutaraldeyde activation).

Glyoxyl agarose [107] has been described for a long time as a very suitable tool to get a very strong enzyme-support multipoint covalent attachment. The support is prepared by etherification of the primary hydroxyl groups of the support with glycidol to introduce diols, that are later oxidized with sodium periodate to get the glyoxyl group [108], as shown in Figure 8, path (a).

This support has several advantages to produce an intense multipoint covalent attachment. 17–20 glyoxyl groups may be introduced by 1000 Å2 [107]. Such a high density of reactive groups implies high possibilities of an intense enzyme-support reaction. Glyoxyl groups have low steric hindrance towards the enzyme-support reaction, and high stability at alkaline pH values (making easy long term storage and even long term enzyme-support multi-interaction even at alkaline pH values) [100]. However, the most important advantage is something that may look at first glance even as a drawback: the imino bond is very weak and reversible. For this reason, this immobilization method only fixes the protein to the support when several enzyme-supports bonds are formed [109]. This means that it is able to direct the enzyme immobilization by the richest area in reactive groups of the protein, enabling very high stabilization at relatively low cost in terms of activity. However, this method has two problems: immobilization requires to be performed at alkaline pH or using some imino-bond stabilizing reagent [110] and a final reduction with sodium borohydride is required to make the bonds irreversible (secondary amino bonds) and transform the remaining aldehyde groups into inert hydroxyl moieties. Borohydride, necessary to obtain a fully inert surface, even under mild conditions may produce some problems in enzymes especially sensible like those bearing a metal ion in the active center, or disulfide bridges very exposed to the medium; in these cases, a strict control of the amount and mainly of the concentration of the reagent must be considered [107].

For example, this allowed to check the distribution of two co-immobilized dehydrogenases by controlling the immobilization rate, and the best distribution was a homogeneous mixture of both enzymes; on the contrary, lower efficiency was observed when one enzyme was forming a crown in the outer section of the pores of the support while the other enzyme was within the core.

These considerations permitted to conclude that each immobilized preparation generates some specific conformations, and that the different inactivation conditions produce inactivation following different inactivation paths, driving to different inactivated structures. This may explain why some immobilization protocols involving some specific enzyme areas may be more efficient in improving enzyme stability under some conditions than under other conditions, while for other orientations the effect is the opposite [111,112]. The changes occurring during immobilization may be very varied. For example, if we are able to froze a hyperactivate state of an enzyme, we can get a improve activity, as occur with the immobilization of lipases on hydrophobic supports that can stabilize the open for of lipase [113]. However, usually the distortions caused on the enzyme structure during immobilization should be expected to cause a decrease of enzyme activity, although some exceptions may be found [114]. And for enzymes that require to experience deep structural changes to express activity, only if the changes are produced before multipoint covalent attachment is accomplished, some activity may be expected to survive. The topic has been extensively reviewed, also with large discussion about agarose-based supports [35].

Starting from glyoxyl or epoxy agarose, it is possible to introduce by incubation with a concentrated solution of ethylenediamine a secondary and a primary amino group on the support surface (agarose-MANE, Figure 8, path (b)). The primary amino group has an special interest, as its pK_a_ is lower than 7, and that makes the support compatible with the immobilization of enzymes via their carboxy groups using the carbodiimide route [115], (Figure 8 path (c)). This enzyme immobilization method shows as main problems that it needs modifying the whole protein surface, and the low stability of the reactive groups arising from carboxy functions and carbodiimide, that may decrease the multipoint covalent attachment even although the number of aspartate (Asp) and glutamate (Glu) usually is the most frequent among the reactive groups of the protein surface [116].

Again starting from glyoxyl or epoxy agarose, carboxy derivatives could be easily prepared by using aminoacids such as 6-aminohexanoic acid. Also in this case the carbodiimide route will be useful, although only primary amino groups of the protein may react with the support, and some enzyme have scarce lysine (Lys) groups in their surface or an exposed terminal amino group. Moreover, an intense multipoint covalent attachment is hardly achieved because of the high pK_a_ of the exposed Lys residues [105].

An alternative used in many instances in enzyme immobilization is the agarose-glutaraldehyde support, easily obtained by modification of agarose-MANE (or other agarose support having primary amino groups) with glutaraldehyde. This group is a very versatile reagent that permits to immobilize an enzyme via diverse orientations and chemistries. First, it is possible to activate the support with one or two molecules of glutaraldehyde, with very different reactivity [117]. Second, it is possible to use pre-activated supports or add the glutaraldehyde after enzyme ionic exchange on agarose-MANE [118]. Due to the heterofunctional nature of the support, the first immobilization cause may be: ion exchange, hydrophobic adsorption, or covalent immobilization. This permits to get enzyme preparations with different properties using the same support, just changing the immobilization conditions. Using lipase, interfacial activation versus the hydrophobic layer of glutaraldehyde cycles on the support permits a new lipase orientation. A deeper discussion on these immobilization protocol may be found in the paper by Barbosa et al. [119].

Epoxy-agarose is not recommended for enzyme immobilization. This is mainly due to the fact that epoxy groups on the agarose support, even though they are in principle able to react with many functional groups of the proteins [120], only have a reasonable reactivity with thiol groups [121]. To obviate the low reactivity of epoxy supports, most of the enzymes are immobilized on commercial epoxy supports via a two-step procedure, i.e., a first hydrophobic enzyme adsorption and a further enzyme-support covalent reaction [37,122,123,124]. The hydrophilic nature of agarose also makes such a hydrophobic adsorption very unlikely. The concept of heterofunctional supports has reverted the situation [125]. Following this idea, some groups able to adsorb proteins (ionic groups, immobilized metal chelates, thiol groups, any other adsorbent) may be introduced on the epoxy supports, and now epoxy-agarose beads can be used for protein immobilization.

From agarose-epoxy, it is possible to get different modified supports. Octyl-agarose is perhaps one of the most successfully employed for protein immobilization, specifically for lipase immobilization. The octyl layer permits the lipase interfacial activation (the mechanism of action of lipases) [126,127,128,129,130] and in that way, this support permits the one step immobilization, purification and stabilization of lipases [131] thanks to the immobilization involving the open form of the lipases [113].

The introduction of other groups to reinforce the lipase immobilization on octyl-agarose has proved to be very useful to improve enzyme stabilization [132,133,134,135]. These octyl-heterofunctional lipase supports may be used in media containing high cosolvent concentrations [136,137] and even in the presence of substrates/products with detergent properties [138].

Agarose activated with vinylsulfone groups has been long known, because divinylsulfone is useful also for cross-linking agarose beads (see Section 3), but only recently the good properties of this group to get an intense multipoint covalent attachment have been revealed [139]. This group is very stable even at alkaline pH value, can react with different groups in the proteins (histidine (His), Lys, cysteine (Cys), tyrosine (Tyr)), its reactivity is high at alkaline pH value and reasonable at neutral and even acid pH, there is not steric hindrance for the enzyme-support reaction, and may also permit immobilization of an enzyme following different orientations [140]. The final blocking step may further alter enzyme properties [141]. This kind of support has proven to overpass the possibilities of producing a very intense multipoint covalent attachment of agarose-glyoxyl. The main drawback is the relatively large spacer arm that reduces the rigidity induced by the multipoint covalent attachment [114,139].

## 5. Outstanding Examples of Enzymes Immobilized on to Agarose-Based Supports

As previously discussed, agarose is one of the most inert polymers that the researcher may use. Therefore, this support is largely used in gel filtration, as enzyme-support interactions are unlikely in most cases, and therefore the enzymes are separated just because of the molecular size [142,143].

This property converts agarose in the ideal support for assaying new strategies for enzyme immobilization, because the only groups that can react with the proteins are those introduced by us, and we can strictly control the enzyme-support interactions.

Immobilized enzymes on agarose beads have been proposed for industrial applications: for example, a thermostable 𝛼-amylase was immobilized by physical entrapment within agarose beads and then applied to clean starch stains from clothes [144]. Another industrially relevant biocatalyst is the FAD-dependent phenylacetone dehydrogenase, which has been successfully immobilized on amino-agarose beads, previously functionalized through covalent attachment of a FAD analogue: the apoenzyme recognized the coenzyme analogue, and their interaction resulted in a fully active reconstituted holoenzyme [145]. Another interesting application deals with the covalent reversible immobilization of *Trametes villosa* laccase, previously reduced and thiolated, on a particular agarose derivative, i.e., thiolsulfinate agarose. The immobilized enzyme is noticeably stable and active, and can find application in bioremediation of wastewaters, contaminated by textile dyes [146].

Moreover, in this section, we will present some of the newest and complex immobilizations of enzymes, assayed taking advantage of this feature.

### 5.1. Systems for Enzyme Co-Immobilization

In many cases the transformation we are developing involves several reactions where the product of one enzyme is the substrate of the other, and the product of interest is the product of the last enzyme [147,148]. These reactions usually require the simultaneous use of several enzymes under the same conditions, an already complex fact because we need to have good enough activity/stability for all involved enzymes. In that way, these so-called cascade reactions make compulsory to use enzymes presenting a range of operational conditions where all of them may be used.

In cascade reactions, the co-immobilization of the enzymes on porous supports offer some kinetic advantages, as the later enzymes may act on high concentrations of the product of the previous enzymes (due to diffusion limitations) [149]. In that way, the lag-time that usually occurred in this process may be avoided. However, after this lag-time, the reaction course may be similar to the use of individually immobilized enzymes, and the kinetic advantages are not actually reflected by the difference in initial reaction rates, but analyzing the whole reaction course [29]. However, co-immobilization may be the only alternative if the product of one of the enzymes is unstable and this lag time may be enough to produce its alteration [29,150,151]. In any case, before deciding to use a co-immobilized enzyme instead of the individually immobilized enzymes, it should be considered that standard co-immobilization has some strong drawbacks: the enzymes are immobilized on the same particles and after inactivation of the less stable enzyme, both enzymes need to be discarded [29,33]. Other problems may arise from the selection of the support (that should have pores large enough for the largest enzyme) [33].

Agarose beads have been used to solve these problems by two different strategies. In the first one, agarose was derivatized to present two kinds of groups, immobilized metal chelate (IMAC) and glyoxyl groups. This heterofunctional support [125] enabled to immobilize two dehydrogenases used to produce dihydroxyacetone from glycerol [152]. One of the enzymes could be stabilized by immobilization on glyoxyl groups, while the other could not be immobilized on this support without suffering large decrements on enzyme activity. The authors solved the situation immobilizing the first enzyme on glyoxyl groups at alkaline pH value, and then the second one (having a poly-His tag) on the IMAC groups. In this way, each enzyme was immobilized on the same surface, but using different chemistries. They did not analyze the half-life of each enzyme, but in case the enzyme immobilized on the IMAC was less stable than the other, this enzyme could be desorbed after inactivation and fresh enzyme added to maintain the ‘combi’ enzyme preparation. This strategy is very elegant but the density of the glyoxyl groups is lower than using standard supports and the IMAC groups may produce some steric hindrance for the glyoxyl-enzyme reaction (they will form a layer over the glyoxyl layer), decreasing the stabilizing effects of the immobilization [125].

A second study was specifically directed to solve some of these problems, although the strategy is only valid if one enzyme is more stable than the other, and this enzyme cannot be stabilized via multipoint covalent attachment [153]. The strategy consisted on immobilizing the most stable and stabilizable enzyme using the best strategy (best support, best immobilization conditions), then coating the immobilized enzyme with a poly-ionic polymer (e.g., polyethyleneimine), treatment that could even improve the immobilized enzyme performance [154], and then immobilizing the second enzyme (less stable and not stabilizable) on this “activated enzyme”. When the second enzyme was inactivated, the enzyme is desorbed from the immobilized and more stable enzyme; the immobilized enzyme is coated with fresh polymer and then with more fresh second enzyme. The authors showed that the enzyme adsorbed on ion exchangers become more strongly adsorbed after immobilization [155], making more difficult the enzyme desorption, but after a careful design of the desorption, the first enzyme could be reused for several adsorption/desorption cycles after inactivation of the first enzyme [153].

### 5.2. Studying the Immobilized Enzyme

Agarose is transparent and chemically inert, enabling to perform studies that hardly may be performed using other supports.

For example, agarose permits to identify and quantify the number of multipoint covalent attachments obtained by immobilization on glyoxyl (secondary amino groups) or divinylsulfone supports (ethers, thioethers, secondary amino groups are formed with the reactive amino acids of the enzyme) [84,114,139]. This cannot be performed using epoxy-acrylic supports because after enzyme cleavage in 1 N HCl at 120 °C, the support generated groups able to react with the enzyme moieties making impossible the quantification of the enzyme groups involved in the immobilization [156].

Fluorescence using confocal microscopy [157] has been used to check the distribution of two co-immobilized dehydrogenases by controlling the immobilization rate and permitted to conclude that the best distribution was a homogeneous mixture of both enzymes; on the contrary minor efficiency was observed when enzymes were not uniformly distributed across the same porous surface [152]. Optimal preparations gave better values using low concentration of cofactor even than using the free enzyme.

Moreover, it has permitted following the infrared spectrum of differently immobilized enzymes subjected to different inactivation conditions [60]. The different preparations were analyzed by deconvolution of the amide I region, which provides information about the secondary structure of the protein in terms of α-helixes, β-sheets, β-turns and non-ordered or irregular structures [158]. These permitted to conclude that each immobilized preparation generates some specific conformations, and that the different inactivation conditions produce inactivation following different inactivation paths, driving to different inactivated structures. This may explain why some immobilizations may be more efficient in improving enzyme stability under some conditions, whereas for other orientations the effect is the opposite [111,112]. The changes occurring during immobilization may be very varied. For example, if we are able to ‘freeze’ a hyper-activated state of an enzyme, we can get an improved activity, as occurs with the immobilization of lipases on hydrophobic supports that can stabilize the open form of lipase [113]. However, usually the distortions caused on the enzyme structure along the immobilization procedure cause a decrease of enzyme activity, although some exceptions may be found [114]. And for enzymes that undergo deep structural changes along their catalytic cycle, only if the changes are produced before multipoint covalent attachment are accomplished some activity may be expected. The topic has been extensively reviewed, also with large discussion about agarose-based supports [35].

Fluorescence anisotropy imaging provides a normalized protein mobility parameter that serves as a guide to study the effect of different immobilization parameters (length and flexibility of the spacer arm and multivalence of the protein-support interaction) on the final stability of the supported proteins [159]. Time/spatial-resolved fluorescence determines anisotropy values of supported-fluorescent proteins through different immobilization chemistries, on agarose activated supports have been utilized to show the lower or higher rigidification obtained after enzyme immobilization [160]. Proteins in a more constrained environment correspond to the most thermostable ones, as was shown by thermal inactivation studies.

## 6. Problems and Perspectives

In conclusion, agarose beads are extremely versatile tools for immobilization procedures, both at a laboratory scale or in technological applications. However, their preparation implies some laborious (and expensive) steps, and the wider availability of crude, less expensive agar from *Gracilaria* is counterbalanced by the need of additional chemical treatments in comparison to preparations coming from *Gelidium* seaweeds.

Stiffness of agarose-based beads is an outstanding feature of such preparations, which renders them quite suitable for applications where hydrostatic pressure is high and a good flux though columns or fluidized beads is important. In particular, highly cross-linked beads show an exceptional mechanical stability, and such beads are quite resistant even against turbulent operative conditions and vigorous mechanical stirring. This ensures a high stability of the immobilized enzyme molecules, that are mainly hosted within the pores and therefore protected against any mechanical offence.

Agarose beads can be obtained within a wide range of particle diameters, so one could choose the commercially available preparations most suitable for the particular application required.

As depicted above, the polyhydric nature of the polymer allows a number of derivatization reactions that could be tailor-made in a manner ensuring the best immobilization conditions for any enzyme one chooses, and saving a substantial fraction of the original activity.

As in the past, it is expected that in the future agarose may maintain its role as model support to assay new immobilization protocols to improve enzyme features. Its physical and chemical inertness are features that no other commercial support has. Its high loading capacity for proteins (even 100 mg/g of wet support), its optical properties, make agarose an invaluable material whose properties should be used as a reference in the development of new materials for enzyme immobilization.

## Figures and Tables

**Table 1:** Main agarophytes used for agar production ^1^.

Species	Location
*Gelidium amansii*	Japan, China
*Gelidium cartilagineum*	USA, Mexico, South Africa
*Gelidium corneum*	South Africa, Portugal, Spain, Morocco
*Gelidium liatulum*	Japan
*Gelidium lingulatam*	Chile
*Gelidium pacificum*	Japan
*Gelidium sesquipedale*	Portugal, Morocco
*Gelidiella acerosa*	Japan, India, China
*Gracilaria verrucosa*	Turkey
*Gracilaria dura*	France
*Gracilaria tenuistipitata*	Philippines
*Pterocladia lucida*	New Zealand, Azores
*Pterocladia capilacea*	Egypt, Japan, New Zealand
*Ahnfeltia plicata*	Russia

^1^ Adapted from [66,67,71,73,74].
